# Physics-Guided Active Learning for New Ligand Discovery

**DOI:** 10.1145/3768322.3769025

**Published:** 2025-12-05

**Authors:** Nikhil Dhiman, Dikshant Sagar, Negin Forouzesh

**Affiliations:** Department of Computer Science, California State University, Los Angeles, Los Angeles, California, USA; Department of Computer Science, University of California, Irvine, Irvine, California, USA; Department of Computer Science, California State University, Los Angeles, Los Angeles, California, USA

**Keywords:** Drug discovery, Active Learning, Generative neural networks

## Abstract

Structure-based drug discovery seeks to identify small molecules that bind to protein targets and modulate their function, but current workflows remain slow, costly, and data-limited. While generative models can design ligands from protein structures, they often lack physical grounding and fail to generalize beyond static training data. We propose an active learning framework that generates 3D ligands conditioned on protein pocket geometry and iteratively retrains on the top 10% of candidates, evaluated using a composite score integrating binding affinity and solvation energy. On the refined PDBBind v2019 dataset, our approach yields consistent dataset-wide improvements: median binding affinity increases from −9.3 to −9.7 kcal/mol (compared to −8.7 kcal/mol for reference ligands), median solvation free energy decreases from 1.32 to 0.94 kcal/mol, and ASKCOS scores improve from −2.17e^4^ to −2.05e^4^, reflecting enhanced synthetic feasibility. On the BRD4 benchmark, our GNINA–AMBER hybrid scoring pipeline correctly ranks a known non-binder last, validating scoring robustness. These results demonstrate that physics-guided active learning systematically enhances ligand quality, diversity, and physical plausibility, offering a scalable and generalizable approach for structure-based drug design.

## 1 Introduction

Structure-based drug discovery (SBDD) aims to design small molecules to bind specific protein sites and modulate function. By using 3D pocket geometry, SBDD enables rational ligand design. However, the process remains slow and expensive, taking 10–15 years [5] and over $1.8 billion [4] per drug, with a failure rate exceeding 90%. A major bottleneck is early-stage lead identification, which takes 3–5 years and often produces candidates lacking in affinity, stability, or synthetic feasibility. This challenge is exacerbated by the scale of chemical space, estimates suggest over 10^60^ [17] drug-like molecules are theoretically possible. Even when constrained to a specific protein structure, the task of identifying viable ligands is computationally intractable. Efficient exploration of this space is essential to reduce timelines and increase success rates.

Recent deep learning advances have led to generative models capable of learning complex molecular distributions for de novo ligand design, conditioned on protein binding pocket structures [16]. These models aim to generate chemically valid molecules with favorable docking profiles. However, purely data-driven methods often suffer from limited generalizability due to narrow training data and lack of physical grounding [7]. Consequently, they may yield ligands that are implausible, unstable, or infeasible, an issue for structure-based drug discovery, which relies on precise atomic interactions and physical constraints. Meanwhile, empirical and physics-based methods (e.g., AutoDock Vina [21]), grounded in molecular mechanics and thermodynamics, provide accurate protein–ligand evaluations but are computationally costly and ill-suited for large-scale chemical space exploration requiring rapid screening of thousands of molecules.

The limitations of purely generative and physics-based models, combined with the scarcity of large-scale, high-quality labeled datasets, motivate the use of an active learning framework. Our approach leverages a deep generative model guided by iterative feedback from physics-based evaluations. By selecting top-performing ligands based on structural and energetic criteria, the model is progressively refined without requiring additional labeled data. This closed-loop strategy enables efficient exploration of chemical space and improves generalization, steering generation toward diverse and physically plausible candidates.

To efficiently explore drug-like chemical space under limited data, we implement an active learning framework built around a Conditional Variational Autoencoder Generative Adversarial Network (CVAEGAN) [18]. The model generates 3D ligands conditioned on protein binding pocket geometry and is iteratively refined through feedback from a downstream evaluation pipeline. Each ligand is scored using GNINA [10] for binding affinity and end-point free energy methods in Amber [2] for solvation stability. In each round, high-scoring ligands are added back to the training set, and the model is retrained to progressively shift toward higher-quality chemical space. Hence, our contribution is as follows:

- We extend the CVAEGAN [18] model to generate ligands conditioned on protein binding pocket geometry, and embed it in an active learning loop where the model is iteratively retrained on high-quality ligands identified through feedback.
- Integrated GNINA [10] and Amber [2] to evaluate binding affinity, pose confidence, and stability.

## 2 Methods and Materials

### 2.1 Atom Encoding and Grid-Based Representation

Molecular inputs are encoded following the CVAEGAN Hybrid scheme [18], where each atom is represented as a one-hot vector over 18 physicochemical and structural features, including atom type and bonding properties. These features are voxelized into 48 × 48 × 48 atomic density grids using Libmolgrid [20], with atoms modeled as truncated Gaussians within a 23.5 Å cube. This representation is tailored for 3D convolutional generative models.

Molecule recovery employs the same atom fitting and bond inference strategy: atom types and coordinates are optimized via Libmolgrid’s [20] differentiable gridding to minimize squared grid error, with high-density points refined by gradient descent. OpenBabel [13] finalizes valid molecular structures by inferring bonds and adding hydrogens.

### 2.2 Deep Generative Model

The original CVAEGAN model [18] incorporated physics-based features into the conditional encoder. In contrast, our method removes these features and conditions ligand generation solely on the 3D geometry of the binding pocket via atomic density grids. This decoupling improves **scalability** by eliminating costly computations, enhances **generalization** by avoiding hardcoded biases, and supports **modularity** by shifting physics to post hoc evaluation for efficient and physically plausible selection.

The revised CVAEGAN [18] comprises four components: input encoder, conditional encoder, decoder, and discriminator. The input encoder maps ligand–receptor complexes into a latent distribution, while the conditional encoder extracts receptor pocket features. Their representations are concatenated and decoded into ligand density grids. A 3D CNN discriminator, trained adversarially, improves output realism [1]. The model is optimized with a multi-objective loss including reconstruction [18], KL divergence, steric clash penalty, and Wasserstein loss [1], enabling generation of chemically plausible ligands conditioned on receptor pockets.

### 2.3 Evaluation Metrics

To evaluate ligand quality, we use a dual-metric scoring strategy combining GNINA-predicted binding affinity [10] and Amber-calculated solvation energy [2]. These metrics were selected after comparing multiple evaluators, including AutoDock Vina [21], GNINA [10], DiffDock [3], and AmberTools [2]. Preliminary results (see Section 3.1) showed that GNINA [10] and Amber [2] prioritize different ligand–protein pairs, making them complementary for selecting diverse yet physically plausible candidates.

#### Binding Affinity (GNINA):

We use GNINA [10] to estimate the binding free energy (Δ*G*_bind_) of each generated ligand–receptor pair. GNINA [10] integrates convolutional neural networks into the docking pipeline to improve scoring accuracy over traditional methods.

#### Solvation Energy (Amber):

We compute solvation energy using AmberTools [2] via the GBNSR6 implicit solvent model. This metric serves as a proxy for ligand stability in an aqueous environment, penalizing hydrophobic or poorly solvated structures.

This combined evaluation ensures that selected ligands exhibit both strong predicted binding and favorable solvation profiles, capturing two key aspects of physical viability.

### 2.4 Active Learning Framework

To iteratively improve the generative model and guide it toward producing higher-quality ligands, we implement a feedback-driven active learning (AL) loop. At each iteration, the CVAEGAN model generates candidate ligands for a fixed set of unseen protein targets. These ligands are evaluated using a combined scoring function based on GNINA [10] binding free energy and Amber [2] solvation energy. Both metrics are first normalized via min–max scaling across the ligand batch and then aggregated into a final combined score.

For Active Learning Iteration, we select the top 10% of ligands based on their combined scores. This threshold balances ligand quality and chemical diversity: selecting fewer candidates (e.g., top 1%) may overfit to score noise or outliers, while selecting too many risks including suboptimal ligands that dilute the learning signal. The selected 10% of high-performing ligands are added to the training set and used to retrain the generative model in the next iteration. This selective data augmentation allows the model to progressively refine its internal distribution over chemically plausible and physically viable ligands. To determine convergence, we track the change in average combined score across iterations. The active learning loop is terminated when the mean score improvement falls below a threshold of Δ_score_ < 0.1 for three consecutive rounds. This criterion ensures that the model stops updating once performance plateaus, avoiding overfitting to early top-ranking structures.

This iterative framework enables the model to explore chemical space more efficiently by reinforcing only the most promising candidates, without relying on fixed datasets or static training objectives. The decoupling of generation and evaluation also provides flexibility to swap in new scoring tools or domain-specific objectives, making the pipeline adaptable to other downstream tasks.

### 2.5 Dataset

#### PDBBind:

The PDBBind database [14] is a widely used benchmark in structure-based drug discovery, containing experimentally resolved protein–ligand complexes from the Protein Data Bank (PDB) with associated binding affinities and atomic-resolution 3D structures making it ideal for training 3D structure-aware generative models.

We use the refined set of PDBBind v2019 [14], comprising 2,728 high-quality protein–ligand complexes selected for resolution and structural clarity. Receptors contain 2,500–3,000 heavy atoms and ligands range from 35–50. The dataset is split 80% for training and 20% for evaluation. To assess generalization across diverse targets, the test set is divided into 10 clusters based on receptor size, each with 5 proteins, enabling stratified evaluation across varied pocket sizes and environments.

#### BRD4 Benchmark Set:

The BRD4 dataset [11] is a curated benchmark for evaluating model performance in distinguishing binders from non-binders for BRD4, a cancer-related protein target. It includes 10 ligands, nine confirmed binders from co-crystal structures and one non-binder (ligand 1), docked into apo BRD4 using AutoDock Vina [21].

## 3 Results and Discussion

### 3.1 Preliminary Results

**Figure 2.**
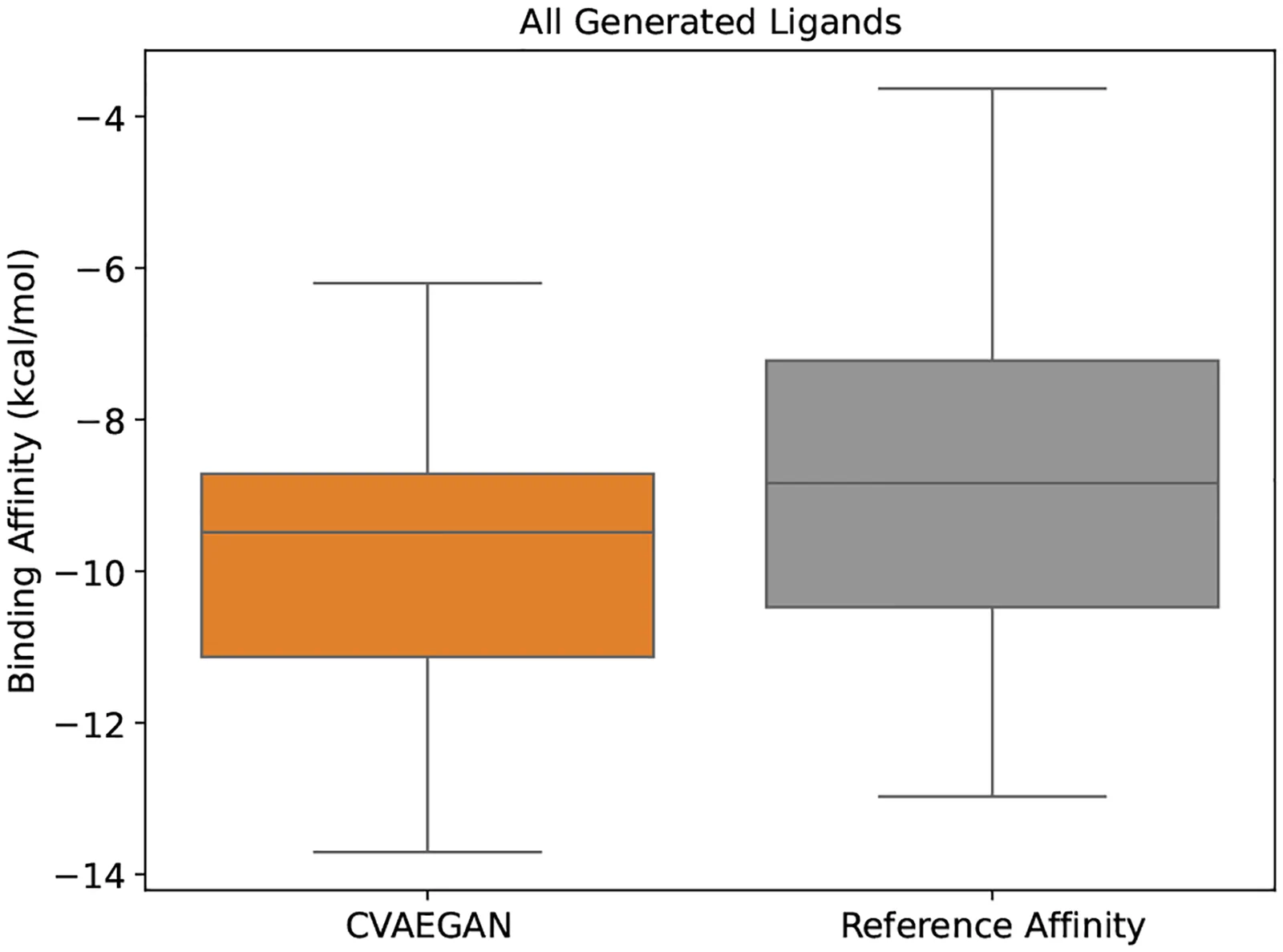
Binding affinities of top-5 CVAEGAN [18] generated ligands vs. reference ligands.

As a baseline, we evaluate CVAEGAN [18] without Active Learning by generating 90 ligands for each of 70 randomly selected proteins. As shown in Figure 2, the generated ligands exhibit a median binding affinity of −9.9 kcal/mol compared to −8.7 kcal/mol for reference compounds, with a narrower spread (−13.7 to −6.5 kcal/mol vs. −12.5 to −4.3 kcal/mol). This indicates that the model produces candidates with consistently stronger predicted binding affinities.

**Figure 3.**
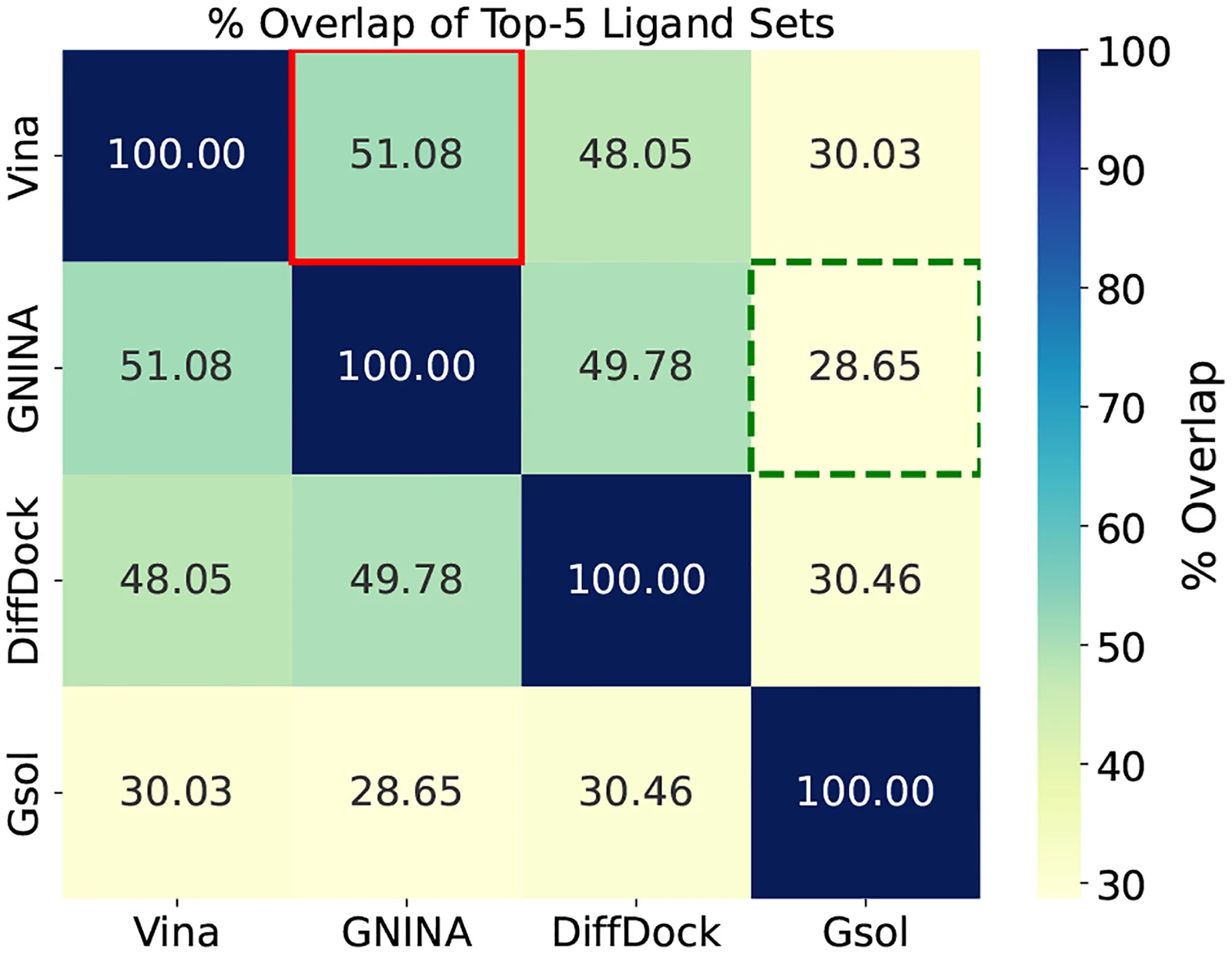
Pairwise overlap of top-5 ligand sets ranked by Vina, GNINA, DiffDock, and Amber (GSol).

To analyze the behavior of different evaluation metrics, we generated a set of 90 ligands for each of 70 randomly selected proteins and selected the top 5 protein–ligand pairs according to each tool: AutoDock Vina [21], GNINA [10], DiffDock [3], and AmberTools [2]. The overlap analysis, shown in Figure 3, reveals moderate agreement between GNINA and DiffDock (highlighted in red), but minimal agreement between GNINA and Amber solvent energy (highlighted in green). Since GNINA emphasizes binding pose and interaction energy while Amber captures solvation and thermodynamic stability, we adopt them as the primary evaluation metrics and combine their scores (see Section 2.3) to account for both affinity and stability while promoting diverse and physically meaningful ligand selection.

### 3.2 BRD4 Benchmark Evaluation

To validate our scoring pipeline, we tested our evaluation matrix on the BRD4 benchmark [11], comprising ligands with experimentally validated binding activity. Complexes were evaluated using GNINA [10] for predicted binding affinity (Δ*G*_bind_) and Amber [2] for solvation free energy (Δ*G*_solv_). Both metrics were first normalized using min–max scaling and then combined into a single Combined Score. As shown in Table 1, binders such as ligand 10 achieved the most favorable scores of 0.93, while ligand 1, the known non-binder, received the least favorable score of 0.38. The GNINA–Amber Combined score effectively discriminates actives from inactives in our active learning loop.

### 3.3 Active Learning Framework Results

To assess the impact of our active learning (AL) framework, we tracked model performance across all refinement iterations. At each round, we clustered proteins into 10 groups based on size and randomly selected 5 proteins from each cluster. For these 50 proteins, the CVAEGAN model generated 10 ligands per protein, yielding 500 candidates per iteration. This sampling strategy balances computational efficiency with representative coverage. The model was subsequently retrained using the top 10% of generated ligands, selected according to a combined score of GNINA [10] predicted binding affinity and Amber [2] calculated solvation energy.

**Figure 4.**
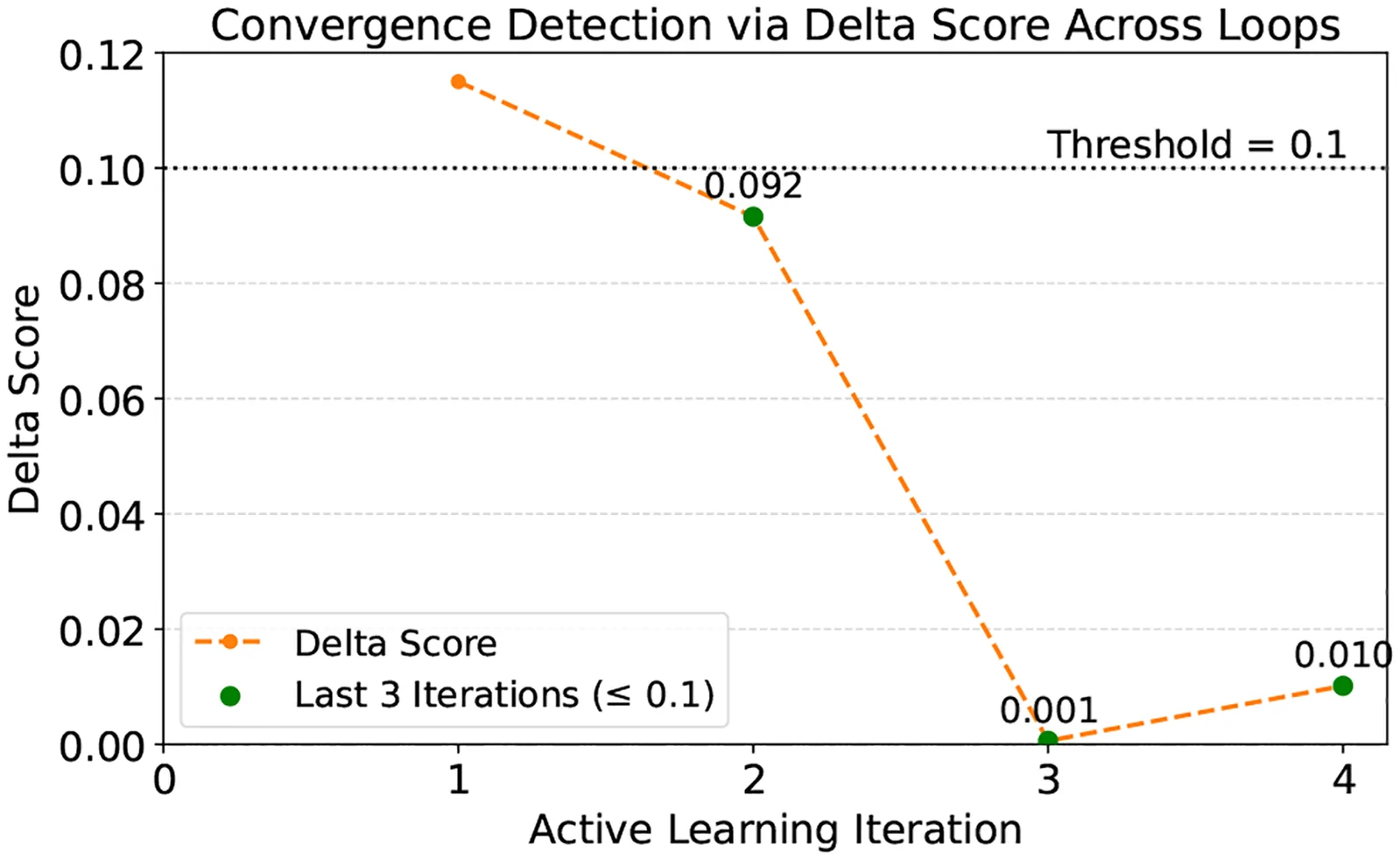
Convergence detection across AL loops. Avg. combined-score change falls below Δ_score_ < 0.1 after loop 3 and remains low (0.010 at loop 4).

As shown in Figure 4, the average change in combined score between iterations decreases sharply over time, indicating effective learning through feedback. By the third iteration, the difference drops to 0.001 and remains low in subsequent rounds (0.010 in iteration 4), consistently below the predefined convergence threshold of Δ_score_ < 0.1 for three consecutive loops. This satisfies our convergence criterion and confirms that the model has stabilized.

**Figure 5.**
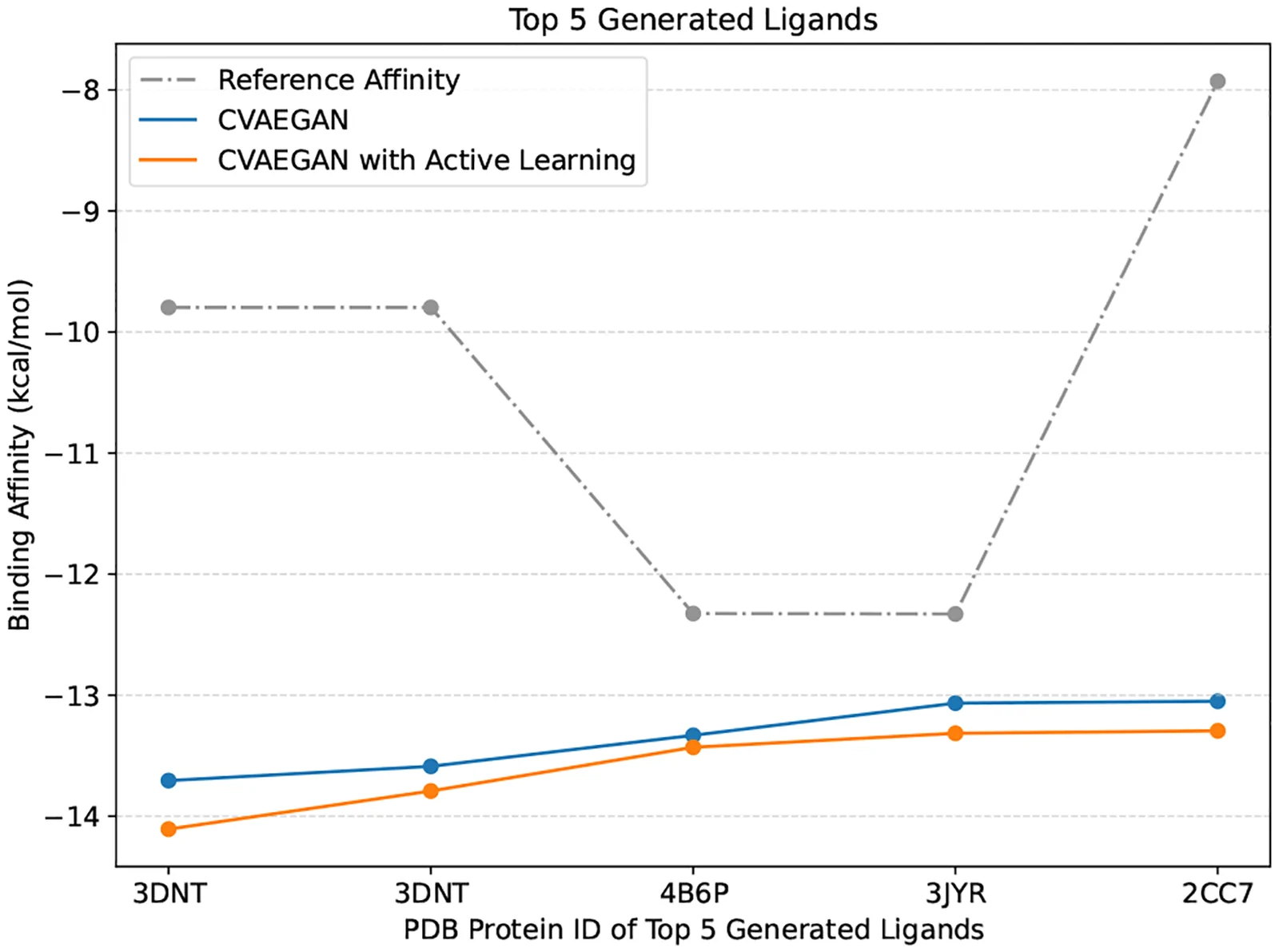
Binding affinity for top-5 generated ligands. CVAEGAN+AL outperforms baseline by 0.401, 0.204, 0.099, 0.249, and 0.244 kcal/mol, respectively, across 3DNT, 4B6P, 3JYR, and 2CC7. Reference ligands scored weaker, ranging from −7.93 to −12.33 kcal/mol.

To evaluate the effect of active learning, we compared the top-5 ligands generated by CVAEGAN with Active Learning against those from the baseline CVAEGAN [18] and their corresponding reference ligands. As shown in Figure 5, CVAEGAN with Active Learning consistently produced stronger binders, improving affinities by 0.401, 0.204, 0.099, 0.249, and 0.244 kcal/mol across the top-5 ranks. For example, CVAEGAN with Active Learning achieved −14.11 vs. −13.71 kcal/mol for 3DNT, −13.79 vs. −13.59 for 2CC7, and −13.43 vs. −13.33 for 4B6P. In contrast, reference ligands were substantially weaker, ranging from −7.93 (2CC7) to −12.33 kcal/mol (4B6P, 3JYR). These results demonstrate that active learning systematically refines ligand generation, yielding more favorable Δ*G*_bind_ values across diverse protein targets.

**Figure 6.**
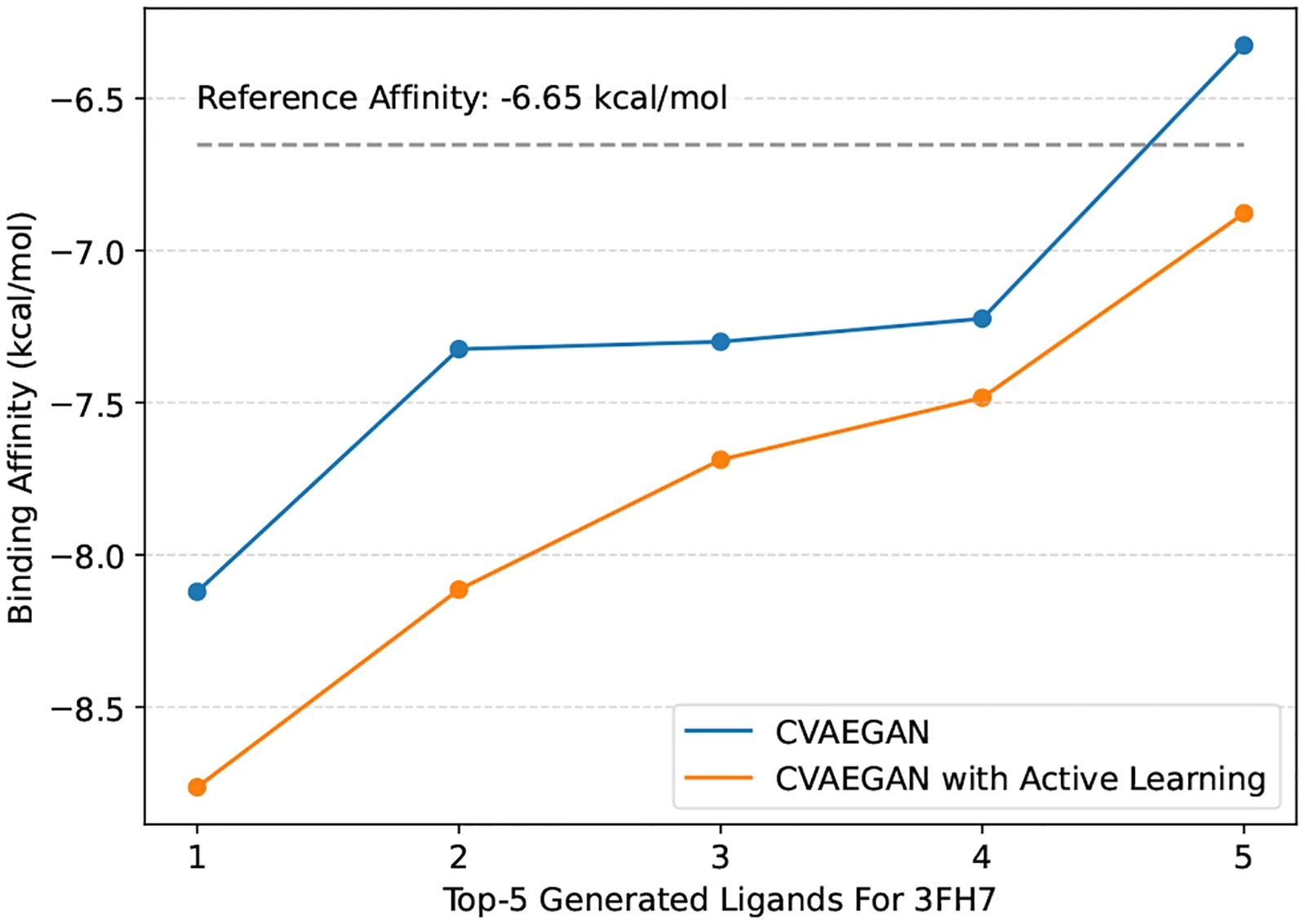
Binding affinity for top-5 ligands targeting 3FHB (Poly(ADP-ribose) polymerase 3). CVAEGAN+AL outperforms baseline by 0.642, 0.790, 0.388, 0.260, and 0.552 kcal/mol, respectively.

We conducted case studies on two therapeutically relevant targets: 3FHB (poly(ADP-ribose) polymerase 3) and 4ASE (Crystal structure of VEGFR2), which illustrate the best and worst per-target ligand quality under active learning, respectively. For 3FHB, the reference ligand has a predicted binding affinity of −6.65 kcal/mol. As shown in Figure 6, the top-5 ligands from the active learning-enhanced model consistently outperform both the baseline and reference, with affinities as low as −8.75 kcal/mol.

**Figure 7.**
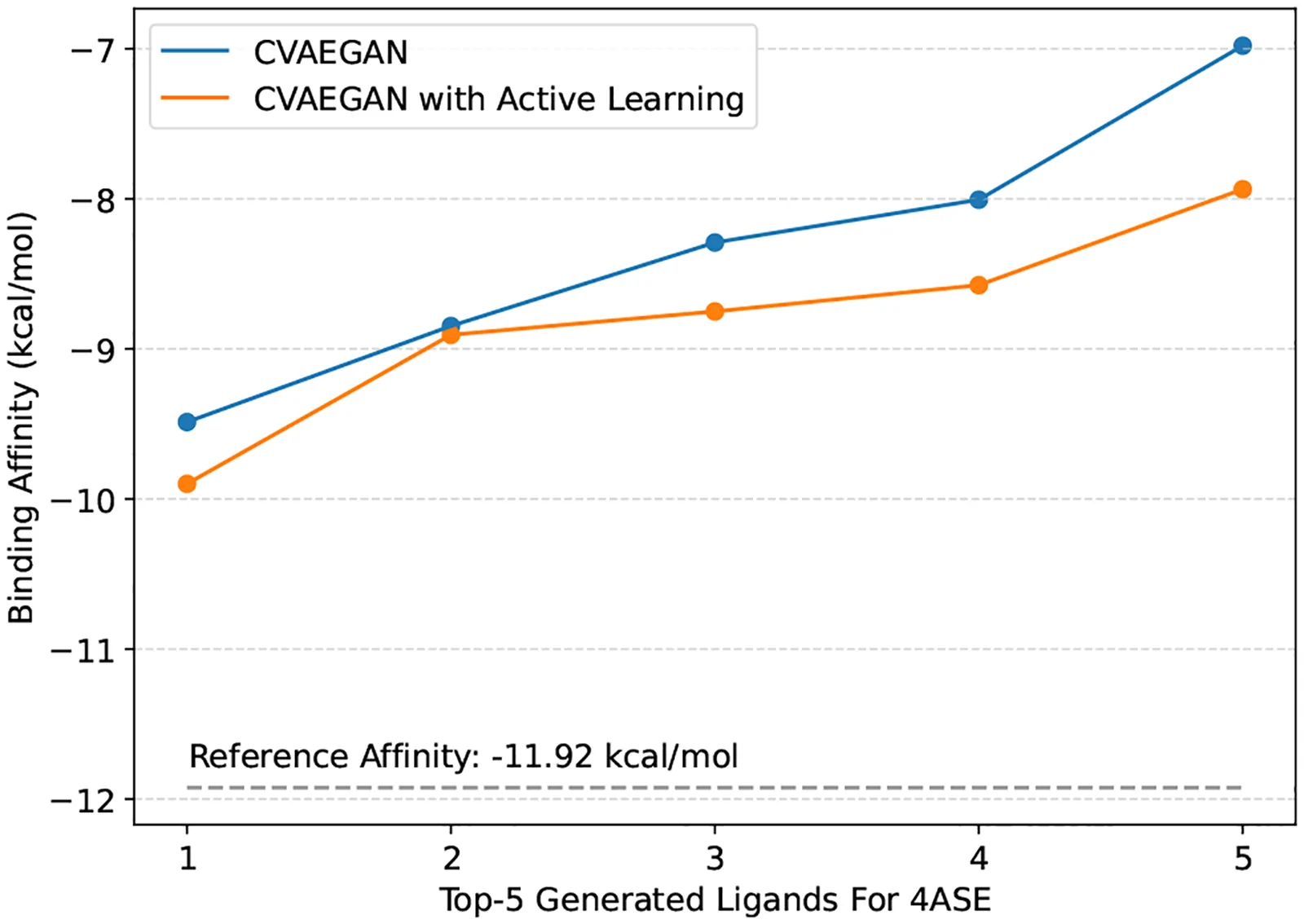
Binding affinity for top-5 ligands targeting 4ASE (Crystal structure of VEGFR2). CVAEGAN+AL performs better than baseline by 0.450, 0.050, 0.250, 0.300, and 0.800 kcal/mol, respectively.

For 4ASE, the reference ligand scores −11.92 kcal/mol. As shown in Figure 7, the active learning enhanced model produces ligands with affinities between −10.0 and −8.0 kcal/mol, consistently stronger than those generated by the baseline CVAEGAN but still weaker than the reference ligand. This result illustrates the benefit of feedback-guided refinement while also indicating that certain targets, such as 4ASE, leave room for further improvement.

**Figure 8.**
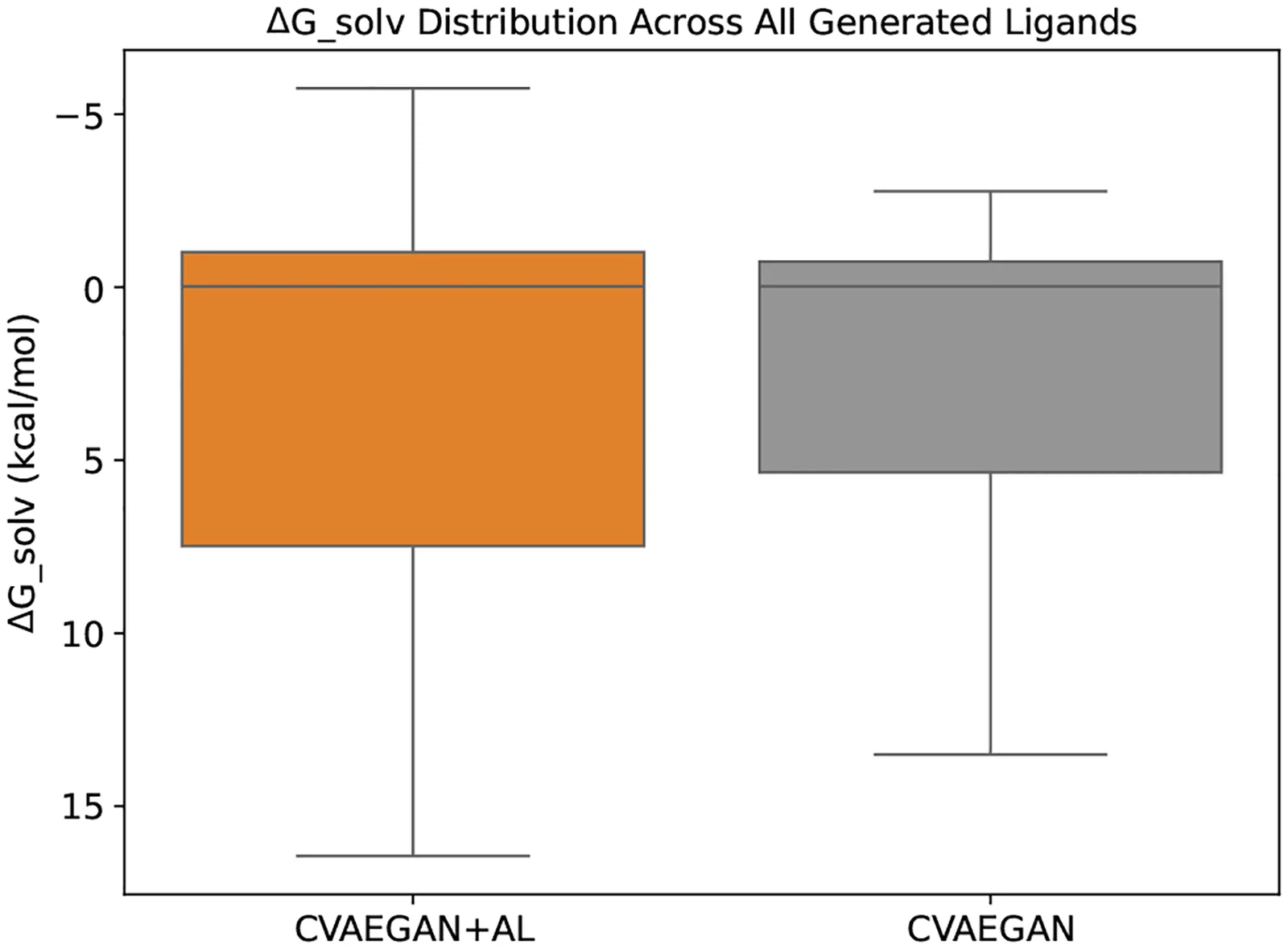
Distribution of solvation free energy (Δ*G*_solv_) for generated ligands under CVAEGAN+AL and CVAEGAN models.

For solvation energy, as shown in Figure 8, CVAEGAN produced ligands with a median Δ*G*_solv_ of 1.32 kcal/mol, while the active learning variant (CVAEGAN+AL) reduced the median to 0.94 kcal/mol. This shift indicates that active learning generates ligands with more favorable solvation properties and broader chemical diversity.

Table 2 reports average molecular metrics across the test set. Compared to the baseline hybrid, CVAEGAN+AL achieves lower binding free energy (−10.82 vs. −10.70). Synthesizability, measured using the ASKCOS retrosynthesis score (−2.05e^4^ vs. −2.17e^4^), improves as less negative values correspond to shorter and more feasible synthetic routes. Key physicochemical descriptors also shift toward drug-like ranges: molecular weight (MW) is slightly reduced, which generally favors absorption and bioavailability; logP, the octanol–water partition coefficient, is higher, reflecting improved lipophilicity and membrane permeability; and hydrogen bond acceptor (HBA) and donor (HBD) counts are lower, reducing excessive polarity that can hinder oral bioavailability. Collectively, these results indicate that active learning not only strengthens binding affinity but also promotes the generation of ligands with more drug-like and synthesizable properties.

## 4 Conclusion

We introduced a physics-guided active learning framework that combines generative modeling with physics-based evaluation to improve ligand discovery. Iterative retraining improved binding affinity, stability, and diversity. While each active learning iteration incurs cost due to physics-based evaluations with GNINA and Amber, these steps are performed offline during training and are not required at inference. Once trained, the model can generate ligands quickly with minimal compute overhead. Future work will extend evaluations of final candidates through molecular dynamics and biochemical analysis to further increase reliability.

## Figures and Tables

**Figure 1.**
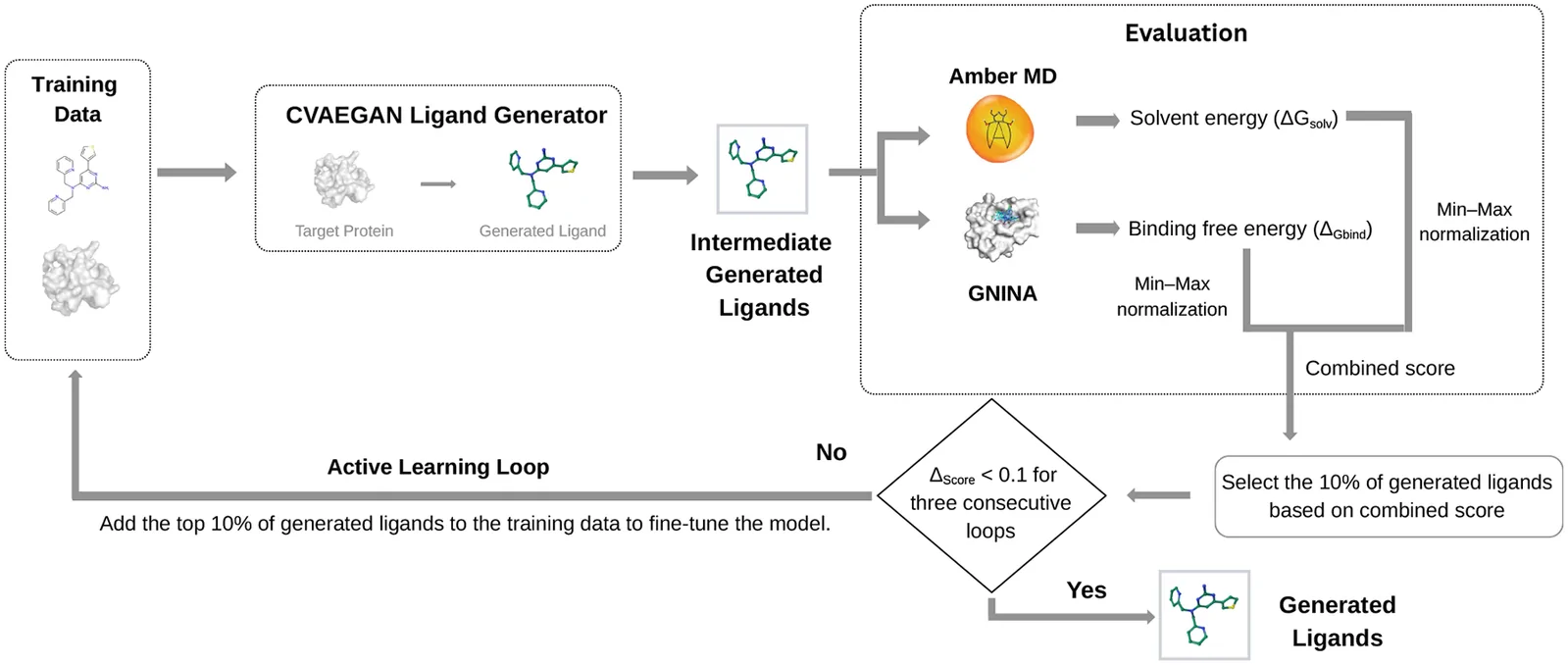
Active learning framework for ligand discovery. The CVAEGAN generates ligands conditioned on target proteins, which are evaluated using Amber (Δ*G*_solv_) and GNINA (Δ*G*_bind_). Normalized scores are combined to rank candidates, with the top 10% iteratively added to retrain the model. Convergence is defined as (Δ_score_) < 0.1 for three successive iterations, yielding the final set of optimized ligands.

**Table 1.** BRD4 validation: GNINA (Δ*G*_bind_), Amber (Δ*G*_solv_), and experimental binding affinities. Binders are ranked higher than the non-binder (Ligand 1).

Lig. ID	Δ*G*_bind_	Δ*G*_solv_	Combined Score	Exp. Δ*G*_bind_
10	−9.40	9.87	0.93	−10.41
5	−9.26	11.59	0.86	−7.40
8	−7.05	8.46	0.64	−8.99
9	−8.25	14.47	0.61	−9.64
6	−6.40	7.87	0.56	−7.84
4	−6.71	10.85	0.51	−6.36
7	−9.34	22.99	0.49	−8.16
**1**	**−5.99**	**11.46**	**0.38**	**NB**
2	−7.36	21.95	0.24	NS

NB: non-binder; NS: no saturation.

**Table 2.** Average binding, synthesizability, and drug-likeness metrics.

Model	Δ*G*_bind_ (↓)	ASKCOS (↑)	m(↓)	LogP	HBA (↓)	HBD (↓)
CVAEGAN Hybrid	−10.70	−2.17e^4^	358.47	−0.35	7.42	4.91
CVAEGAN + AL	−10.82	−2.05e^4^	356.90	−0.25	7.30	4.85

## CCS Concepts:

- **Computing methodologies** → *Modeling methodologies*; *Neural networks*.
